# Cyberchondria severity and utilization of health services in Polish society: a cross-sectional study

**DOI:** 10.1186/s12889-024-18399-9

**Published:** 2024-03-27

**Authors:** Mateusz Kobryn, Mariusz Duplaga

**Affiliations:** Department of Health Promotion and e-Health, Institute of Public Health, Faculty of Health Sciences, Jagiellonian University Medical College, Skawinska Str. 8, 31-066 Krakow, Poland

**Keywords:** Cyberchondria, Health services, Health anxiety, Chronic disease, Disability, COVID-19 vaccination

## Abstract

**Background:**

It has been suggested that cyberchondria leads to increased utilization of healthcare services. Unfortunately, not many studies have analyzed this effect comprehensively. The aim of this study was to analyze the relationship between cyberchondria severity and the utilization of healthcare services among adult Internet users after adjusting for sociodemographic characteristics and the health status of respondents.

**Methods:**

The analysis detailed in this paper examined data from a computer-based, web-based interviewing survey performed among a representative sample of 1613 Polish Internet users. Cyberchondria severity was assessed with the Cyberchondria Severity Scale (CSS). The variables reflecting the use of healthcare services were based on the frequency of visits to family physicians and specialists, diagnostic procedures, hospital admissions, and emergency services, and finally being vaccinated against COVID-19. The effect of cyberchondria severity on the utilization of healthcare services and alternative medicine was adjusted for key sociodemographic variables, the presence of chronic diseases, disability, and unspecific symptoms. For variables reflecting the use of services, ordinal logistic regression and multivariable logistic regression models were developed.

**Results:**

Cyberchondria severity was a significant predictor of the utilization of all but one of the analyzed healthcare services and alternative medicine. The odds of being in a higher category of the utilization of visits to family physicians and specialists, hospital admissions, emergency services and alternative medicine services increased by a factor of 1.01–1.02 for every unit increase of the cyberchondria score. The cyberchondria score was a negative predictor of COVID-19 vaccine uptake. The effect of cyberchondria on outcome variables was independent of the level of health anxiety, sociodemographic variables, and variables reflecting the health status of respondents.

**Conclusions:**

Cyberchondria leads to more intensive use of nearly all healthcare services, but in the case of vaccination against COVID-19, cyberchondria severity was a negative predictor. Cyberchondria’s effect extends beyond health anxiety.

**Supplementary Information:**

The online version contains supplementary material available at 10.1186/s12889-024-18399-9.

## Background

Cyberchondria consists in ‘an excessive and/or repeated online health search that is associated with increased distress or health anxiety and persists despite interference with functioning and negative consequences’ [1]. Health anxiety in people with cyberchondria increases as a result of repetitive searches for health information online instead of decreasing. Significantly, the concerns over one’s health escalate with the frequency of such activities [2].

Cyberchondria has raised a growing interest in recent years. Initially, it was triggered by the rapidly increasing use of online health information in modern societies. The COVID-19 pandemic was another stimulus for studies on cyberchondria, particularly in the context of the misinformation flooding the Internet and its potential role in augmenting health anxiety [3, 4]. Conversely, cyberchondria was also considered a factor responsible for vulnerability to developing anxiety during the pandemic [5].

The term health anxiety is used by many authors interchangeably with hypochondriasis. As a result, cyberchondria is sometimes considered a form of hypochondriasis. Although cyberchondria and health anxiety or hypochondriasis overlap, the relationship between both conditions is not fully clear. In the 5th Edition of the Diagnostic and Statistical Manual of Mental Disorders (DSM-5), the term hypochondriasis was substituted with ‘illness anxiety disorder,’ defined as a preoccupation with having or acquiring a serious illness [6]. In the case of cyberchondria, it is underlined that emotional distress is mainly associated with health-related Internet use [1]. However, cyberchondria is not included in the classification of mental disorders and is not treated as a formal diagnosis.

Although a considerable number of studies have been reported about cyberchondria so far [7] and several tools have been proposed to measure it [8], many areas still require explanation. The available studies have clearly shown that there is a positive correlation between health anxiety and seeking online health information, as well as between health anxiety and cyberchondria [9]. It is assumed that people with high levels of health anxiety turn to the Internet hoping for relief, and they become addicted to repetitive searches for health-related content. However, they are not relieved; on the contrary, their health anxiety increases. Therefore, cyberchondria may be perceived as a type of vicious cycle in which health anxiety leads to excessive searching for health information online. Still, this activity does not lead to lowered anxiety but to its further augmentation.

To what extent the lack of reliable and reassuring health resources adds to this vicious cycle is unclear. It seems we also lack sufficient evidence to understand the roles of health and ehealth literacy in the development of cyberchondria. While some studies have shown that, contrary to expectations, e health literacy may be positively associated with cyberchondria severity [10, 11], Savkin et al. did not observe a significant association between health literacy and cyberchondria in healthcare workers from Turkey [12]. Finally, Yousefi et al. reported a significant negative relationship between cyberchondria and information literacy in medical science students [13].

Some authors have suggested that cyberchondria may be related to increased utilization of health care services. However, a closer analysis of available evidence does not clearly show such a relationship. Initial studies have suggested that online health information searches may lead to the deterioration of the doctor-patient relationship [14]. McElroy et al. suggested that online health searches, the main feature of cyberchondria, may lead to increased healthcare costs due to visits to multiple doctors, aka ‘doctor shopping’ [15]. Another argument for claims that cyberchondria results in increased utilization of healthcare resources was an observation that people who frequently searched for health information on the Internet proceeded to queries about local healthcare services [2].

Cyberchondria resulting in repeated requests for consultations, and hence, increased healthcare utilization was also considered by Vismara et al. [16]. McElroy et al. claimed that a significant proportion of healthcare costs and productivity losses associated with medically unexplained symptoms may be related to cyberchondria [15]. According to their reasoning, cyberchondria is related to increased health anxiety, and the latter, according to some reports, represents a significant economic burden for the healthcare system; therefore, cyberchondria is responsible for increased costs in the healthcare system [15]. A review of papers reporting at least some results of analyzing the relationship between the utilization of healthcare services and cyberchondria shows that unequivocal evidence of such a relationship is unavailable, with the reviewed studies providing inconsistent results. Some authors reported such a relationship [17] others did not [18]. Some authors found significant correlations between subscores of the cyberchondria severity scale and utilization of health services but not with the total cyberchondria score [19]. It should be noted that these studies differed in study samples and measures used for assessing the utilization of health services.

It may be surprising that among the many studies of cyberchondria, only a few addressed its interaction with the healthcare system. In this study, we aim to analyze the relationship between the level of cyberchondria and the utilization of various types of health services using data from a computer-based web interviewing (CAWI) survey of a representative sample of Polish Internet users. We also want to ascertain whether cyberchondria is a predictor of using so-called ‘alternative medicine.’ We hypothesize that a higher level of cyberchondria is associated with more frequent use of family physician and specialist services, diagnostics procedures, and vaccination against COVID-19. Another hypothesis we would like to test is that cyberchondria leads to more frequent use of emergency services and hospital admissions. Finally, we hypothesize that higher intensity of cyberchondria is also related to more frequent use of alternative medicine.

## Materials and methods

### Survey

The analysis reported in this paper was conducted on data from a CAWI survey performed among a representative sample of 1613 Internet users in Poland, aged 18–75. The survey was carried out over ten days from April 1–10, 2022, by the PBS limited liability company (Sopot, Poland) [20] among the participants of an Internet panel maintained by this company [21]. The structure of the study sample was adjusted for gender, age, level of education, place of residence, and Nomenclature of Territorial Units for Statistics (NUTS) region. The quota was established from data reported by Statistics Poland, the main statistical office in Poland [22]. For the sample of 1613 respondents, assuming the size of the population of adult Internet users in Poland at about 24,000,000, a fraction of 0.5, and a confidence level of 0.95, the sampling error was less than 2.5%. The survey data were used earlier to analyze the antecedents of cyberchondria among adult Internet users in Poland [23].

The study received the consent of the Bioethical Committee of Jagiellonian University (decision No KBET/107/B/2011 amended September 8, 2021). The research team obtained an anonymized dataset from the PBS Company. No personal data enabling the identification of respondents were processed during the analysis.

### Questionnaire

The questionnaire used in the survey consisted of 108 items, including the Cyberchondria Severity Scale (CSS) [15, 24]; the 16-item European Health Literacy Questionnaire (HLS-EU-Q16) [25]; the eHealth Literacy Scale [26, 27]; the Short Health Anxiety Inventory (SHAI) [28]; a set of questions asking about the use of the Internet and social media, the presence of chronic diseases, unexplained symptoms and disabilities, the utilization of healthcare services; and questions about sociodemographic variables. In this analysis, apart from the cyberchondria score calculated based on the CSS, we applied two SHAI subscores, calculated for the perception of illness likelihood (SHAI-IL) and predicting negative consequences (SHAIP-NC), and variables reflecting health status (presence of chronic diseases and presence of unexplained symptoms) and prevalence of disabilities, as well as the age and gender of respondents.

### Measures

The utilization of healthcare services was measured with five categorical variables asking about the number of episodes of specific healthcare services used by a respondent in the last two years (“How many times did you use following services in the last two years?”). Only one of these variables was dichotomous; the remaining could assume three or four values. The variables indicating visits to family physicians and specialists and undergoing diagnostic procedures had four categories (0– no utilization, 1– once, 2– two to three times, 3– more than three times). The variables indicating emergency visits and hospital admissions had three categories (0– no utilization, 1– once, 2– more than once). The variable measuring the use of alternative medicine services also had three possible values. Finally, the variable reflecting COVID-19 vaccination was dichotomous (0– no vaccination, 1– vaccination).

The total cyberchondria severity score was calculated based on the Cyberchondria Severity Scale, consisting of 30 items with response options ranging from ‘never’ to ‘always’ transformed to numerical values ranging from 1 to 5, respectively [24]. The score could range from 30 to 150. According to Bajcar et al., the instrument was characterized by high internal consistency (Cronbach alpha = 0.95). The Cronbach alpha coefficient calculated for our sample assumed the same value as reported by Bajcar et al. [24]. The SHAI was used to calculate two subscores; the first based on the subscale expressing the perception of illness likelihood (SHAI-IL), which consisted of 14 items and could range from 0 to 42, and the second related to predicted negative consequences (SHAI-NC) including four items and resulting score assuming values from 0 to 12 [28]. Cronbach alpha coefficients reported for the Polish version of the SHAI scale surpassed 0.90 [28].

### Statistical analysis

The statistical analysis was conducted using the IBM SPSS v.28 (IBM Corp. Armonk, NY, USA) software. Mean and standard deviations (SD) were calculated for continuous numerical variables. Categorical variables were statistically described with absolute and relative frequencies.

Differences in the cyberchondria score depending on the frequency of the utilization of healthcare services and the use of alternative medicine were analyzed based on nonparametric tests; the Kruskall-Wallis test for the variable expressing the utilization of services, assuming more than two options; and the U Mann-Whitney test for the variable showing the uptake of COVID-19 vaccination. In the case of significant values in the Kruskall-Wallis test, post hoc paired tests were conducted to check the differences between categories of the frequency of the services used after applying Bonferroni correction.

Multivariate ordinal regression models were developed for the variables reflecting the utilization of healthcare services and with values representing three or four ordered categories. For dichotomous variable showing the uptake of COVID-19 vaccination, a multivariable logistic regression model was developed. In each model, the cyberchondria score, SHAI-IL, SHAI-NC, age, gender, the presence of chronic diseases, unexplained symptoms, and disabilities were included as independent variables.

Multicollinearity was tested for ordinal logistic regression and logistic regression models. Model fit was assessed with the likelihood-ratio test. The goodness of fit was checked with Pearson and deviance chi2 tests. Cox and Snell, Nagelkerke, and McFadden R2 approximation coefficients were calculated for ordinal logistic regression models. The proportional odds assumption for the ordinal logistic regression models was checked with the test of parallel lines. As this test is anti-conservative and leads to the rejection of proportional odds assumptions in the case of large samples, a large number of explanatory variables, and the inclusion of continuous variables in the model [29, 30], the p-value was set for this test at < 0.01. The Hosmer-Lemeshow test and the Nagelkerke R2 coefficient were calculated for the logistic regression model. P-values, odds ratios (OR), and 95% confidence intervals were reported for the independent variables included in the regression models. P-values lower than 0.05 were deemed to be significant.

## Results

### Characteristics of the study group

In the study sample, 48.7% were men (Table 1). Respondents with at least one chronic disease made up 37.1% (*n* = 607), and those complaining of unexplained symptoms made up 21.1% (*n* = 340). 11.8% (*n* = 190) of the study group were people with disabilities. The mean age (SD) of respondents was 42.0 (14.2), the mean SHAIP-NC score was 2.8 (2.5), and the mean SHAIP-IL score was 11.7 (6.9). The cyberchondria score in the study group was 69.1 (20.5).

**Table 1:** Characteristics of the study group [continuous variables are summarized by mean (standard deviation) and categorical variables by frequency (percentage)]

	Variables	M (SD) or n (%)
Demographics	Age	42.0 (14.2)
	Gender	
	- Female	828 (51.3)
	- Male	785 (48.7)
Health status	Chronic diseases	
	- No	867 (53.8)
	- Yes	607 (37.1)
	Unexplained symptoms	
	- No	1273 (78.9)
	- Yes	340 (21.1)
	Disability	
	- No	1423 (88.2)
	- Yes	190 (11.8)
	SHAIP-NC score	2.8 (2.5)
	SHAPI-IL score	11.7 (6.9)
	Cyberchondria severity score	69.1 (20.5)
Utilization of healthcare services	Visits to family physicians	
	- no visits	215 (13.3)
	- one visit	244 (15.1)
	- two or three visits	505 (31.3)
	- more than three visits	649 (40.3)
	Visits to specialists	
	- no visits	355 (22.0)
	- one visit	289 (17.9)
	- two or three visits	450 (27.9)
	- more than three visits	519 (32.2)
	Diagnostic procedures	
	- no use	458 (28.4)
	- once	342 (21.2)
	- two or three times	441 (27.3)
	- more than three times	372 (23.1)
	Hospital admission	
	- no admission	1184 (73.4)
	- one admission	266 (16.5)
	- more than one admission	163 (10.1)
	Emergency services	
	- no use	1130 (70.1)
	- once	291 (18.0)
	- more than once	192 (11.9)
	Uptake of COVID-19 vaccination	
	- not vaccinated	603 (37.9)
	- vaccinated	987 (62.1)
	Alternative medicine	
	- no use	1363 (84.5)
	- once	89 (5.5)
	- more than once	161 (10.0)

Abbreviations: M– mean, SD– standard deviation, SHAIP-NC - the Short Health Anxiety Inventory– Illness Likelihood subscale, SHAI-NC - the Short Health Anxiety Inventory– Negative Consequences subscale

### Analysis of cyberchondria scores by categories of healthcare services utilization

Univariate analysis revealed that the cyberchondria severity score differed significantly depending on the frequency of utilization of all healthcare and alternative medicine services (Table 2). In the case of all variables reflecting the use of services, apart from COVID-19 vaccination, the cyberchondria score was lower in subgroups that less frequently used specific services. Respondents who were not vaccinated against COVID-19 had significantly higher cyberchondria scores.

The difference between mean cyberchondria scores among respondents who did not attend family physician visits in the last two years and those who attended them more than three times was 8.6. In the case of the groups with the lowest and the highest frequencies of visits to specialists, the difference in mean cyberchondria scores was 9.2, and in the case of such groups regarding diagnostic procedures, the difference was 4.6. In turn, the difference in mean cyberchondria scores between groups with no hospitalization and with more than one hospitalization the difference of mean score was as high as 12.2. In the case of groups of respondents who did not use and used emergency services more than once, the difference was 11.9. Finally, the difference of mean cyberchondria scores between groups of respondents not using and using alternative medicine more than once in the last two years was as high as 11.1.

**Table 2:** Univariate nonparametric analysis of cyberchondria severity depending on the frequency of utilization of healthcare services and alternative medicine

Variable	Variable category	Mean (SD)	p
Visits to family physicians	no visits	63.2 (20.6)^ab^	H = 32.72, df = 3, *p* < 0.001
	one visit	66.5 (19.8)^c^	
	two or three visits	69.3 (19.9)^a^	
	more than three visits	71.9 (20.8)^bc^	
Visits to specialists	no visits	63.2 (19.5)^abc^	H = 32.99, df = 3, *p* < 0.001
	one visit	68.8 (19.1)^a^	
	two or three visits	70.2 (21.0)^b^	
	more than three visits	72.4 (20.7)^c^	
Diagnostic procedures	no use	66.7 (20.6)^a^	H = 19.80, df = 3, *p* < 0.001
	once	68.8 (19.5)^b^	
	two or three times	69.2 (20.1)^c^	
	more than three times	71.3 (20.4)^abc^	
Hospital admission	no admission	67.0 (19.7)^ab^	H = 50.41, df = 2, *p* < 0.001
	one admission	72.1 (20.4)^ac^	
	more than one admission	79.2 (23.2)^bc^	
Emergency services	no use	66.5 (19.4)^ab^	H = 63.25, df = 2, *p* < 0.001
	once	73.4 (20.3)^ac^	
	more than once	78.4 (23.9)^bc^	
Alternative medicine	no use	67.4 (19.8)^ab^	H = 54.97, df = 2, *p* < 0.001
	once	79.4 (20.6)^a^	
	more than once	77.9 (22.5)^b^	
Uptake of COVID-19 vaccination	not vaccinated	71.3 (20.8)	U=-3.621, *p* < 0.001
	vaccinated	67.6 (20.2)	

p - p-value for U Mann-Whitney test in the case of the dichotomous grouping variable and the Kruskal-Wallis test for grouping variables with more than two categories; H– Kruskall-Wallis test statistics; U– U Mann-Whitney test statistics, df– degrees of freedom; a, b, c,– the same letter shows categories of variables significantly different in posthoc test after applying Bonferroni correction

### Determinants of utilization of healthcare services

Cyberchondria severity was a significant predictor in all but one of the ordinal regression models developed for response variables reflecting the utilization of healthcare services and alternative medicine (Table 3). For one unit increase of the cyberchondria score response variable was expected to change by 1.01–1.02, all other variables in the model held constant. Only the use of diagnostic procedures was not significantly associated with cyberchondria severity (OR, 95%CI: 1.00, 1.0-1.01). Detailed results of ordinal regression modeling are available in Additional File 1.

**Table 3:** Ordinal regression modeling of variables reflecting the utilization of healthcare services and alternative medicine

Variable	Variable category	Visits to family physicians	Visits to specialists	Diagnostic procedures	Hospital admission		Emergency services	Alternative medicine	
		OR (95%CI)	OR (95%CI)	OR (95%CI)	OR (95%CI)		OR (95%CI)	OR (95%CI)	
Age		1.00 (0.99-1.01)	0.99 (0.985-0.998)	1.02 (1.01-1.02)	0.98 (0.98-0.99)		0.98 (0.97-0.99)	0.99 (0.98-1.00)	
CSS score		1.01 (1.001-1.012)	1.01 (1.00-1.01)	1.00 (1.00-1.017)	1.01 (1.01-1.02)		1.02 (1.01-1.02)	1.02 (1.01-1.03)	
SHAI_IL (illness likelihood)		1.07 (1.05-1.09)	1.05 (1.03-1.08)	1.08 (1.06-1.10)	1.04 (1.021-1.06)		1.04 (1.01-1.06)	1.02 (0.99-1.04)	
SHAI-NC (negative consequences)		0.89 (0.85-0.94)	0.94 (0.90-0.98)	0.92 (0.88-0.97)	0.98 (0.93-1.03)		0.97 (0.92-1.02)	1.03 (0.97-1.10)	
Gender	male#								
	female	1.11 (0.92-1.33)	1.44 (1.20-1.73)	1.27 (1.06-1.52)	1.12 (0.89-1.41)		0.84 (0.67-1.04)	1.01 (0.76-1.32)	
Presence of chronic disease(s)	yes#								
	no	0.35 (0.28-0.44)	0.30 (0.24-0.37)	0.31 (0.25-0.38)	0.53 (0.41-0.69)		0.64 (0.50-0.82)	0.68 (0.49-0.93)	
Unexplained symptoms	yes#								
	no	0.72 (0.56-0,92)	0.80 (0.63-1.02)	0.74 (0.59-0.94)	0.99 (0.75-1.30)		0.73 (0.56-0.95)	0.92 (0.66-1.28)	
Disabilities	yes#								
	no	0.85 (0.62-1.18)	0.58 (0.42-0.80)	1.04 (0.77-1.40)	0.43 (0.31-0.60)		0.50 (0.36-0.70)	0.77 (0.51-1.17)	
Model fitting	chi^2^	278.40	331.93	376.93	149.29	162.23		74.10	
	p-value	<0.001	<0.001	<0.001	<0.001	<0.001		<0.001	
Goodness of fit test	Pearson chi^2^	4942.74	4853.83,	4915.53	3103.98	3119.39		3237.05	
	pvalue	0.122	0.394	0.186	0.920	0.887		0.389	
Model summary	Nagelkerke R^2^	0.17	0.20	0.22	0.11	0.12		0.07	
Parallel lines test	chi^2^	19.84	22.33	26.83	15.25	17.56		10.86	
	p-value	0.228	0.133	0.043	0.054	0.025		0.210	

# - reference category, OR– odds ratio, 95%CI– 95% confidence interval

The SHAI-Il subscore was a significant predictor of all variables reflecting the utilization of healthcare services but not alternative medicine services (Table 3). The odds ratios indicated that the odds of being in a higher category of utilization of healthcare services increased by a factor of 1.04–1.08 for every unit increase of SHAI-IL. SHAI-NC score was a significant negative predictor of visits to family physicians and specialists, as well as the use of diagnostic procedures.

Age was significantly associated with the frequency of the utilization of healthcare services apart from visits to family physicians and the use of alternative medicine (Table 3). The odds of being in a higher category of utilization of visits to specialists, hospital admissions and emergency services decreased by a factor of 0.98–0.99 for every year of age. In turn, the odds of being in a higher category of utilization of diagnostic procedures increased by a factor of 1.02 for every year of age. The odds of being in a higher category of the frequency of visits to specialists (OR, 95%CI: 1.44, 1.2–1.73) and diagnostic procedures (OR, 95%CI: 1.27, 1.06–1.52) was higher for women than for men.

Respondents without chronic diseases showed lower odds of being in a higher level of the variable reflecting the frequency of visits to family physicians (OR, 95%CI: 0.31, 0.25–0.38) and specialists (OR, 95%CI: 0.30, 0.24–0.37), and the utilization of diagnostic procedures (OR, 95%CI: 0.31, 0.25–0.38), emergency services (OR, 95%CI: 0.64, 0.50–0.82), and hospital admissions (OR, 95%CI: 0.53, 0.41–0.69) (Table 3). They also had a lower probability of being in a higher category of the variable indicating the utilization of alternative medicine (OR, 95%CI: 0.68, 0.49–0.93). Respondents without unexplained symptoms had lower odds of being classified to the higher category of the variable reflecting the utilization of visits to family physicians (OR, 95%CI: 0.72; 0.56–0.92), diagnostic procedures (OR, 95%CI: 0.74, 0.59–0.94), and emergency services (OR, 95%CI: 0.73, 0.56–0.95). In turn, persons without disabilities had lower odds of being in a higher category of the variable reflecting the frequency of visits to specialists (OR, 95%CI: 0.58, 0.42–0.80), hospital admissions (OR, 95%CI: 0.43, 0.31–0.60), and emergency services (OR, 95%CI: 0.50, 0.36–0.70).

The determinants of uptake of COVID-19 vaccination were analyzed with a multivariable logistic regression model (Table 4). An increase of the cyberchondria severity score by 1 point was significantly associated with a decrease in the odds ratio of undergoing vaccination by a factor of 0.991, all other variables in the model being held constant. In turn, an increase of the SHAI-IL score by 1 point was associated with an increase in the odds ratio of vaccine uptake by a factor 1.03. Finally, older respondents were also more likely to undergo vaccination (OR, 95%CI: 1.03, 1.03–1.04).

**Table 4:** Multivariable logistic regression models of uptake of COVID-19 vaccination

Variable	Variable category	B	SE	Wald chi2	OR (95%CI)	p	
Age		0.03	0.004	63.65	1.03 (1.03–1.04)	< 0.001	
CSS score		-0.01	0.003	7.55	0.991 (0.985–0.998)	0.006	
SHAI_IL (illness likelihood)		0.03	0.01	5.83	1.03 (1.01–1.05)	0.016	
SHAI-NC (negative consequences)		-0.04	0.03	2.16	0.96 (0.91–1.01)	0.141	
Gender	female#						
	male	0.13	0.11	1.53	1.14 (0.92–1.41)	0.216	
Presence of chronic disease(s)	yes#						
	no	-0.15	0.13	1.33	0.86 (0.67–1.11)	0.249	
Unexplained symptoms	yes#						
	no	-0.11	0.14	0.63	0.89 (0.68–1.18)	0.428	
Disabilities	yes#						
	no	0.004	0.19	0.0004	0.98 (0.70–1.44)	0.984	
Hosmer & Lemeshow test		chi^2^ = 9.68. p-value = 0.288					
Omnibus test of model coefficients		chi^2^ = 109.59. p-value < 0.001					
Model summary		Nagelkerke R^2^ = 0.09					

# - referential category, OR– odds ratio, 95%CI– 95% confidence interval

## Discussion

The analysis of data obtained from a CAWI survey among a representative sample of adult Internet users in Poland showed that the severity of cyberchondria is a significant predictor of all but one of the variables reflecting the utilization of healthcare services, as well as the use of alternative medicine. Overall, an increase of the cyberchondria score by 1 point was associated with an increased likelihood (not exceeding 2%) of classification to the group more frequently attending family physicians and specialists, using emergency services, and being admitted to the hospital in ordinal logistic regression models. These results confirm our initial hypotheses. We have also observed that the cyberchondria score is a negative predictor of the uptake of COVID-19 vaccination. Our hypothesis assumed that the relationship between these variables would be positive.

Interest regarding research on cyberchondria was heightened during the COVID-19 pandemic. However, as Zheng suggested, research on cyberchondria is still in its ‘infancy’ period [7]. Various theoretical frameworks have been proposed for cyberchondria [16]. From empirical research, we know that there is a relationship between intensified online searches for health information and health anxiety, and in turn, augmented health anxiety is significantly associated with cyberchondria [8]. It is also obvious that cyberchondria differs from common online health information searches.

Some authors also implied that cyberchondria may lead to unfavorable consequences such as the impairment of mental health and lower quality of life [31]. Furthermore, it was also suggested that an unsatisfied need for health information may lead to so-called ‘doctor shopping’ or increased ordering of medical products online. Surprisingly, broader evidence for such effects of cyberchondria is not available [7, 31]. Also, only a few studies addressed so far the association between the utilization of healthcare services and cyberchondria severity. Our study was designed to fill this gap in knowledge about cyberchondria.

Analysis of Polish Internet users shows that utilizing nearly all types of healthcare services is significantly associated with cyberchondria severity. However, other authors have rarely included variables reflecting the utilization of healthcare services in their studies of cyberchondria. Barke et al. reported in 2016 that cyberchondria was moderately correlated with healthcare utilization measured by the number of visits to GP and other health professionals during the preceding year [17].

Mathes et al. reported that cyberchondria was strongly associated with functional impairment separate from health anxiety but not with decreased quality of life [19]. They also found that the overall cyberchondria score was not significantly associated with healthcare utilization, but that the domain of ‘reassurance seeking’ of the cyberchondria scale was positively correlated with physical and mental health service utilization. Furthermore, the ‘excessiveness’ domain was negatively associated with using mental health services.

Recently, Satyarup et al. reported no significant difference in the cyberchondria severity between respondents who had or hadn’t had medical or dental check-ups in the last year in a sample of information technology professionals [18]. Our sample, apart from a different geographical location, was representative of the whole population of Polish adult Internet users.

Some authors suggest that cyberchondria is related to mistrust of healthcare systems. Following this reasoning, one could expect that those with greater cyberchondria severity will avoid using healthcare services and instead use more Internet searches. It seems that our study does not confirm this assumption. Satyarup et al. observed that greater cyberchondria severity was significantly associated with the fear of visits to a doctor or dentist, but in their study, this fear did not result in lower attendance to medical or dental check-ups [18].

One could also speculate that mistrust of the healthcare system may lead to repeated visits to physicians, resulting in the above-mentioned “doctor shopping.” In our study, respondents with more severe cyberchondria were more likely to not only visit family physicians and specialists but also to use emergency services and be hospitalized. It also seems that the relationship between cyberchondria and mistrust of health professionals needs further research. Although some authors tend to associate cyberchondria with mistrust of health professionals, the authors of the cyberchondria measuring tool have reported that the subscale assessing mistrust should not be included in the overall score showing the severity of cyberchondria [15].

In our analysis, the effect of cyberchondria on the utilization of health services is adjusted by the level of health anxiety, as measured with the SHAI tool. We decided to apply separately, in developed regression models, the subscores based on two subscales of the SHAI scale showing the perception of illness likelihood and negative consequences in line with the suggestions of the author who adapted the instrument to Polish [28]. The relationship between cyberchondria and health anxiety is rather complex, and according to earlier reports, cyberchondria is characterized by positive feedback between online health information searches and health anxiety, resulting in their reciprocal augmentation. Our results seem to confirm the suggestion indicating that cyberchondria is a more complex phenomenon than health anxiety. Although the SHAI-IL subscore was positively associated with the utilization of all analyzed healthcare services (but not alternative medicine), we observed an independent effect of cyberchondria on the utilization of all healthcare services apart from the use of diagnostic procedures. The size of this effect may be illustrated by the differences between mean cyberchondria severity scores calculated for the extreme categories of the variables reflecting the utilization of healthcare services. These differences spanned from 4.6 for the use of diagnostic procedures to 12.2 for admissions to the hospital at the maximum range of the cyberchondria severity score of 30–150.

The lack of a significant relationship between cyberchondria score and the use of diagnostic procedures in the ordinal regression model is itself very interesting and counterintuitive. Simple reasoning about the consequences of cyberchondria could lead to the notion that people anxious about their health and trying to find health information from digital sources would also be more prone to demand additional diagnostic work-ups from healthcare providers. Apparently, the main role in increasing such demand is played by health anxiety.

The overall trend for a positive relationship between cyberchondria and the utilization of healthcare services was disturbed by the negative effect of cyberchondria on the uptake of COVID-19 vaccination. In his paper suggesting that the Internet may add to the decreased credibility of healthcare systems, Radwan indicated cyberchondria, misinformation, and infodemic as phenomena potentially responsible for such effects [32]. Our findings that cyberchondria severity is a negative predictor of the uptake of COVID-19 vaccination seem to support Radwan’s assertion. We had earlier observed a high dependence of one’s attitude toward COVID-19 vaccination on extra-medical circumstances, such as political sympathies and conspiracy beliefs in Polish society [33, 34]. People experiencing more severe cyberchondria may be more extensively exposed to political propaganda and conspiracies propagated on the Internet due to longer time devoted to online searches. As a result, they would be more susceptible to health denialism, taking radical forms concerning the COVID-19 pandemic and vaccination on the Internet [35].

Our study revealed that cyberchondria is a predictor of the utilization of healthcare services after adjusting for factors significantly associated with utilizing such services, including chronic disease, unexplained symptoms, or disabilities. Independent of the effect of cyberchondria, these three variables were significantly associated with at least some of the dependent variables reflecting the utilization of healthcare services. We have also adjusted the role of cyberchondria for age and gender - sociodemographic variables indicated as significantly associated with the frequency of healthcare service utilization [36, 37]. Interestingly, multivariate models, including those for cyberchondria, and variables related to health status, revealed that older persons are less willing to visit specialists or emergency services and are less often admitted to the hospital. Age was positively associated only with the frequency of undergoing diagnostic procedures.

We have also found that more intense cyberchondria is significantly related to using alternative medicine. The propensity for non-evidence-based interventions among persons with cyberchondria was also observed by Baspinar [38]. She reported that persons who take nutritional supplements have significantly higher cyberchondria scores. The relationship between cyberchondria and the acceptance of non-academic medical approaches was not addressed sufficiently in the literature. Our study shows that respondents are not necessarily particularly discriminative in selecting types of health services, and alternative medicine interventions seem to them as legitimate as the services provided within academic medicine.

### Limitations

The cross-sectional design of the study does not allow for cause-reason relationship analysis. We observed a consistent effect of cyberchondria on the utilization of healthcare services, but using data from the CAWI survey alone, we cannot explore the responsible mechanisms.

The study was based on the respondents’ perception of their usage of healthcare services, and their responses could be subject to bias, influencing the analyzed relationships.

Due to the extent of the survey questionnaire and many addressed aspects, we were not able to include more items exploring in detail the utilization of healthcare services. We also had to resign from including potentially relevant items to analyze the utilization of healthcare services, e.g., more detailed medical history or previous experience in interactions with the healthcare system.

We were also not able to include other interesting issues related to health behaviors, e.g., the propensity to self-medicate, the use of dietary supplements, or interactions with other consumers of health information on the Internet.

## Conclusions

To our knowledge, this is the first analysis performed on a large representative sample of adult Internet users, and it shows that the severity of cyberchondria is significantly associated with the utilization of healthcare services. We have also confirmed that the cyberchondria score predicts the use of alternative medicine. Our study supports suggestions appearing in earlier papers considering the consequences of cyberchondria in relation to interaction with the healthcare system. The key role of online health information searches in cyberchondria is also associated with greater susceptibility to the misinformation that flooded the Internet during the COVID-19 pandemic, resulting in mistrust in the recommended preventive measures for COVID-19.

We agree there are still many areas related to cyberchondria requiring further research. The relationship between mistrust of health professionals and cyberchondria is one of them. The attitude toward the reliability of online health information among people experiencing cyberchondria is another. Finally, the mechanism responsible for the more intense utilization of healthcare resources in cyberchondria also needs more research.

### Electronic supplementary material

Below is the link to the electronic supplementary material.

Supplementary Material 1

## Data Availability

The datasets used and/or analyzed during the current study are available from the corresponding author upon reasonable request.
