# Supplementary material for: Cyberchondria severity and utilization of health services in Polish society: a cross-sectional study
Source: BMC Public Health. 2024 Mar 27;24:902. doi: 10.1186/s12889-024-18399-9 (PMC10967182; doi:10.1186/s12889-024-18399-9)
Supplement: Supplementary file 1 — Supplementary Material 1 [file 12889_2024_18399_MOESM1_ESM.docx]

**Supplementary table 1 Detailed results of ordinal regression modeling of the variable reflecting the use of visits to family physicians**

| **Variable** | **Categories of variable** | **Estimate** | **SE** | **Wald test** | **df** | **95%CI** | | **p-value** | |  |
| --- | --- | --- | --- | --- | --- | --- | --- | --- | --- | --- |
|  |  |  |  |  |  | **LL** | **UL** |  | | |
| **Threshold** | **[FAM = ,00]** | -2.14 | 0.31 | 46.70 | 1 | -2.75 | -1.53 | <.001 |  |  |
|  | **[FAM = 1,00]** | -1.11 | 0.31 | 12.78 | 1 | -1.72 | -0.5 | <.001 |  |  |
|  | **[FAM = 2,00]** | 0.39 | 0.31 | 1.56 | 1 | -0.22 | 0.99 | 0.212 |  |  |
| **Age** |  | 0.001 | 0.004 | 0.003 | 1 | -0.01 | 0.01 | 0.956 |  |  |
| **CSS score** |  | 0.01 | 0.003 | 5.55 | 1 | 0.001 | 0.01 | 0.019 |  |  |
| **SHAI_IL (illness likelihood)** |  | 0.07 | 0.01 | 43.94 | 1 | 0.05 | 0.09 | <.001 |  |  |
| **SHAI-NC (negative consequences)** |  | -0.11 | 0.02 | 21.71 | 1 | -0.16 | -0.07 | <.001 |  |  |
| **Gender** | **male#** |  |  |  |  |  |  |  |  |  |
|  | **female** | 0.10 | 0.10 | 1.11 | 1 | -0.09 | 0.29 | 0.292 |  |  |
| **Presence of chronic disease(s)** | **yes#** |  |  |  |  |  |  |  |  |  |
|  | **no** | -1.05 | 0.11 | 84.87 | 1 | -1.28 | -0.83 | <.001 |  |  |
| **Unexplained symptoms** | **yes#** |  |  |  |  |  |  |  |  |  |
|  | **no** | -0.33 | 0.13 | 6.73 | 1 | -0.59 | -0.08 | 0.009 |  |  |
| **Disabilities** | **yes#** |  |  |  |  |  |  |  |  |  |
|  | **no** | -0.16 | 0.17 | 0.92 | 1 | -0.48 | 0.17 | 0.337 |  |  |

FAM – variable reflecting the use of visits to family physician, # - reference category, SE – standard error, df – degrees of freedom, LL – lower limit, UL – upper limit

**Supplementary table 2. Detailed results of ordinal regression modeling of the variable reflecting the use of visits to specialists.**

| **Variable** | **Categories of variable** | **Estimate** | **SE** | **Wald test** | **df** | **95%CI** | | **p-value** |
| --- | --- | --- | --- | --- | --- | --- | --- | --- |
|  |  |  |  |  |  | **LL** | **UL** |  |
| **Threshold** | **[SPEC = .00]** | -2.057 | 0.306 | 45.062 | 1 | -2.658 | -1.456 | <.001 |
|  | **[SPEC = 1.00]** | -1.084 | 0.304 | 12.708 | 1 | -1.681 | -0.488 | <.001 |
|  | **[SPEC = 2.00]** | 0.278 | 0.303 | 0.841 | 1 | -0.316 | 0.871 | 0.359 |
| **Age** |  | -0.009 | 0.003 | 6.231 | 1 | -0.015 | -0.002 | 0.013 |
| **CSS score** |  | 0.008 | 0.003 | 8.628 | 1 | 0.003 | 0.013 | 0.003 |
| **SHAI_IL (illness likelihood)** |  | 0.052 | 0.010 | 27.125 | 1 | 0.033 | 0.072 | <.001 |
| **SHAI-NC (negative consequences)** |  | -0.064 | 0.024 | 7.293 | 1 | -0.110 | -0.017 | 0.007 |
| **Gender** | **male#** |  |  |  |  |  |  |  |
|  | **female** | 0.363 | 0.093 | 15.171 | 1 | 0.180 | 0.545 | <.001 |
| **Presence of chronic disease(s)** | **yes#** |  |  |  |  |  |  |  |
|  | **no** | -1.208 | 0.112 | 116.206 | 1 | -1.427 | -0.988 | <.001 |
| **Unexplained symptoms** | **yes#** |  |  |  |  |  |  |  |
|  | **no** | -0.224 | 0.124 | 3.268 | 1 | -0.467 | 0.019 | 0.071 |
| **Disabilities** | **yes#** |  |  |  |  |  |  |  |
|  | **no** | -0.540 | 0.162 | 11.114 | 1 | -0.857 | -0.222 | <.001 |

SPEC – variable reflecting the use of visits to specialists, # - reference category, SE – standard error, df – degrees of freedom, LL – lower limit, UL – upper limit

**Supplementary table 3. Detailed results of ordinal regression modeling of the variable reflecting the use of diagnostic procedures**

| **Variable** | **Categories of variable** | **Estimate** | **SE** | **Wald test** | **df** | **95%CI** | | **p-value** |
| --- | --- | --- | --- | --- | --- | --- | --- | --- |
|  |  |  |  |  |  | **LL** | **UL** |  |
| **Threshold** | **[DIAG = .00]** | -0.505 | 0.297 | 2.895 | 1 | -1.086 | 0.077 | 0.089 |
|  | **[DIAG = 1.00]** | 0.562 | 0.297 | 3.587 | 1 | -0.020 | 1.144 | 0.058 |
|  | **[DIAG = 2.00]** | 2.037 | 0.300 | 46.031 | 1 | 1.448 | 2.625 | <.001 |
| **Age** |  | 0.015 | 0.003 | 20.311 | 1 | 0.009 | 0.022 | <.001 |
| **CSS score** |  | 0.002 | 0.003 | 0.469 | 1 | -0.003 | 0.007 | 0.494 |
| **SHAI_IL (illness likelihood)** |  | 0.076 | 0.010 | 58.334 | 1 | 0.056 | 0.095 | <.001 |
| **SHAI-NC (negative consequences)** |  | -0.080 | 0.024 | 11.669 | 1 | -0.127 | -0.034 | <.001 |
| **Gender** | **male#** |  |  |  |  |  |  |  |
|  | **female** | 0.238 | 0.093 | 6.589 | 1 | 0.056 | 0.420 | 0.010 |
| **Presence of chronic disease(s)** | **yes#** |  |  |  |  |  |  |  |
|  | **no** | -1.182 | 0.110 | 115.424 | 1 | -1.397 | -0.966 | <.001 |
| **Unexplained symptoms** | **yes#** |  |  |  |  |  |  |  |
|  | **no** | -0.297 | 0.121 | 6.037 | 1 | -0.535 | -0.060 | 0.014 |
| **Disabilities** | **yes#** |  |  |  |  |  |  |  |
|  | **no** | 0.036 | 0.154 | 0.056 | 1 | -0.266 | 0.339 | 0.813 |

DIAGN – variable reflecting the use of diagnostic procedures, # - reference category, SE – standard error, df – degrees of freedom, LL – lower limit, UL – upper limit

**Supplementary table 4. Detailed results of ordinal regression modeling of the variable reflecting hospital admissions**

| **Variable** | **Categories of variable** | **Estimate** | **SE** | **Wald test** | **df** | **95%CI** | | **p-value** |
| --- | --- | --- | --- | --- | --- | --- | --- | --- |
|  |  |  |  |  |  | **LL** | **UL** |  |
| **Threshold** | **[HOSP = .00]** | 0.578 | 0.353 | 2.678 | 1 | -0.114 | 1.271 | 0.102 |
|  | **[HOSP = 1.00]** | 1.843 | 0.357 | 26.583 | 1 | 1.142 | 2.543 | <.001 |
| **Age** |  | -0.017 | 0.004 | 15.027 | 1 | -0.025 | -0.008 | <.001 |
| **CSS score** |  | 0.013 | 0.003 | 14.504 | 1 | 0.006 | 0.019 | <.001 |
| **SHAI_IL (illness likelihood)** |  | 0.040 | 0.011 | 11.869 | 1 | 0.017 | 0.062 | <.001 |
| **SHAI-NC (negative consequences)** |  | -0.022 | 0.028 | 0.626 | 1 | -0.078 | 0.033 | 0.429 |
| **Gender** | **male#** |  |  |  |  |  |  |  |
|  | **female** | 0.113 | 0.117 | 0.939 | 1 | -0.116 | 0.342 | 0.332 |
| **Presence of chronic disease(s)** | **yes#** |  |  |  |  |  |  |  |
|  | **no** | -0.627 | 0.133 | 22.372 | 1 | -0.887 | -0.367 | <.001 |
| **Unexplained symptoms** | **yes#** |  |  |  |  |  |  |  |
|  | **no** | -0.013 | 0.142 | 0.008 | 1 | -0.29 | 0.265 | 0.927 |
| **Disabilities** | **yes#** |  |  |  |  |  |  |  |
|  | **no** | -0.843 | 0.169 | 25.001 | 1 | -1.173 | -0.512 | <.001 |

HOSP – variable reflecting hospital admissions, # - reference category, SE – standard error, df – degrees of freedom, LL – lower limit, UL – upper limit

**Supplementary table 5. Detailed results of ordinal regression modeling of the variable reflecting the use of emergency services**

| **Variable** | **Categories of variable** | **Estimate** | **SE** | **Wald test** | **df** | **95%CI** | | **p-value** |
| --- | --- | --- | --- | --- | --- | --- | --- | --- |
|  |  |  |  |  |  | **LL** | **UL** |  |
| **Threshold** | **[EMERG = ,00]** | 0.291 | 0.342 | 0.725 | 1 | -0.379 | 0.961 | 0.394 |
|  | **[EMERG = 1,00]** | 1.543 | 0.345 | 20.020 | 1 | 0.867 | 2.218 | <.001 |
| **Age** |  | -0.020 | 0.004 | 23.484 | 1 | -0.028 | -0.012 | <.001 |
| **CSS score** |  | 0.017 | 0.003 | 26.320 | 1 | 0.010 | 0.023 | <.001 |
| **SHAI_IL (illness likelihood)** |  | 0.034 | 0.011 | 9.346 | 1 | 0.012 | 0.056 | 0.002 |
| **SHAI-NC (negative consequences)** |  | -0.030 | 0.027 | 1.159 | 1 | -0.083 | 0.024 | 0.282 |
| **Gender** | **male#** |  |  |  |  |  |  |  |
|  | **female** | -0.179 | 0.112 | 2.549 | 1 | -0.399 | 0.041 | 0.110 |
| **Presence of chronic disease(s)** | **yes#** |  |  |  |  |  |  |  |
|  | **no** | -0.448 | 0.129 | 12.038 | 1 | -0.701 | -0.195 | <.001 |
| **Unexplained symptoms** | **yes#** |  |  |  |  |  |  |  |
|  | **no** | -0.318 | 0.134 | 5.628 | 1 | -0.582 | -0.055 | 0.018 |
| **Disabilities** | **yes#** |  |  |  |  |  |  |  |
|  | **no** | -0.685 | 0.168 | 16.592 | 1 | -1.014 | -0.355 | <.001 |

EMERG – variable reflecting the use of emergency services, # - reference category, SE – standard error, df – degrees of freedom, LL – lower limit, UL – upper limit

**Supplementary table 6. Detailed results of ordinal regression modeling of the variable reflecting the use of alternative medicine**

| **Variable** | **Categories of variable** | **Estimate** | **SE** | **Wald test** | **df** | **95%CI** | | **p-value** |
| --- | --- | --- | --- | --- | --- | --- | --- | --- |
|  |  |  |  |  |  | **LL** | **UL** |  |
| **Threshold** | **[ALT = .00]** | 2.558 | 0.438 | 34.172 | 1 | 1.701 | 3.416 | <.001 |
|  | **[ALT = 1.00]** | 3.084 | 0.441 | 48.915 | 1 | 2.220 | 3.948 | <.001 |
| **Age** |  | -0.008 | 0.005 | 2.228 | 1 | -0.018 | 0.002 | 0.136 |
| **CSS score** |  | 0.020 | 0.004 | 23.811 | 1 | 0.012 | 0.027 | <.001 |
| **SHAI_IL (illness likelihood)** |  | 0.016 | 0.014 | 1.300 | 1 | -0.011 | 0.043 | 0.254 |
| **SHAI-NC (negative consequences)** |  | 0.030 | 0.033 | 0.813 | 1 | -0.035 | 0.096 | 0.367 |
| **Gender** | **male#** |  |  |  |  |  |  |  |
|  | **female** | 0.005 | 0.142 | 0.001 | 1 | -0.273 | 0.284 | 0.969 |
| **Presence of chronic disease(s)** | **yes#** | 0a | . | . | 0 | . | . | . |
|  | **no** | -0.392 | 0.162 | 5.874 | 1 | -0.709 | -0.075 | 0.015 |
| **Unexplained symptoms** | **yes#** | 0a | . | . | 0 | . | . | . |
|  | **no** | -0.084 | 0.17 | 0.244 | 1 | -0.418 | 0.249 | 0.621 |
| **Disabilities** | **yes#** | 0a | . | . | 0 | . | . | . |
|  | **no** | -0.263 | 0.214 | 1.515 | 1 | -0.681 | 0.156 | 0.218 |

ALT – variable reflecting the use of alternative medicine, # - reference category, SE – standard error, df – degrees of freedom, LL – lower limit, UL – upper limit
